# The effect of opioid-free anesthesia protocol on the early quality of recovery after major surgery (SOFA trial): study protocol for a prospective, monocentric, randomized, single-blinded trial

**DOI:** 10.1186/s13063-021-05829-x

**Published:** 2021-11-27

**Authors:** Maxime Léger, Solène Pessiot-Royer, Tristan Perrault, Elsa Parot-Schinkel, Fabienne Costerousse, Emmanuel Rineau, Sigismond Lasocki

**Affiliations:** 1 Département d’Anesthésie Réanimation, Centre Hospitalier Universitaire d’Angers, Angers, France; 2 INSERM UMR 1246 - SPHERE, Nantes University, Tours University, Nantes, France; 3 Centre Hospitalier Universitaire d’Angers, Département de Biostatistiques et Méthodologie, Angers, France

**Keywords:** Anesthesia, Opioid-free anesthesia, Opioid-related adverse effects, Quality of recovery, Quality of life, Perioperative care, Patient-reported outcome measures

## Abstract

**Background:**

Since the 2000s, opioid-free anesthesia (OFA) protocols have been spreading worldwide in anesthesia daily practice. These protocols avoid using opioid drugs during anesthesia to prevent short- and long-term opioid side effects while ensuring adequate analgesic control and optimizing postoperative recovery. Proofs of the effect of OFA protocol on optimizing postoperative recovery are still scarce. The study aims to compare the effects of an OFA protocol versus standard anesthesia protocol on the early quality of postoperative recovery (QoR) from major surgeries.

**Methods:**

The SOFA trial is a prospective, randomized, parallel, single-blind, monocentric study. Patients (*n* = 140) scheduled for major plastic, visceral, urologic, gynecologic, or ear, nose, and throat (ENT) surgeries will be allocated to one of the two groups. The study group (OFA group) will receive a combination of clonidine, magnesium sulfate, ketamine, and lidocaine. The control group will receive a standard anesthesia protocol based on opioid use. Both groups will receive others standard practices for general anesthesia and perioperative care. The primary outcome measure is the QoR-15 value assessed at 24 h after surgery. Postoperative data such as pain intensity, the incidence of postoperative complication, and opioid consumption will be recorded. We will also collect adverse events that may be related to the anesthetic protocol. Three months after surgery, the incidence of chronic pain and the quality of life will be evaluated by phone interview.

**Discussion:**

This will be the first study powered to evaluate the effect of OFA versus a standard anesthesia protocol using opioids on global postoperative recovery after a wide range of major surgeries. The SOFA trial will also provide findings concerning the OFA impact on chronic pain incidence and long-term patient quality of life.

**Trial registration:**

ClinicalTrials.gov NCT04797312. Registered on 15 March 2021

## Background

Opioids play an essential part in pain control during anesthesia, even in the “balanced anesthesia” concept. However, opioids are associated with many side effects (e.g., nausea, vomiting, ileus, sedation, delirium, addiction, hyperalgesia) [1]. Since the 2000s, opioid-free anesthesia (OFA) protocols have been spreading worldwide in anesthesia daily practice. These protocols avoid using opioid drugs during anesthesia to prevent short- and long-term opioid side effects while ensuring adequate analgesic control and optimizing postoperative recovery.

The emergence of OFA protocols parallels the emergence of an “opioid crisis” (i.e., a dramatic increase in opioid addiction and of related death), particularly in the USA, with a significant proportion of treatment initiation during the perioperative period [2, 3]. This lead scientific societies to reduce patients’ exposure to opioids [4]. OFA is based on a multimodal combination of nonopioid agents such as N-methyl-d-aspartate antagonists, anti-inflammatory drugs, local anesthetics, and alpha-2 agonists. Currently, the anesthetic literature regarding OFA remains controversial, with studies confirming or not its benefit [5–8]. The most recent randomized controlled study published is negative but focused only on opioid-related adverse events [9].

Suggested effects reported in previous studies of multimodal use of OFA are intraoperative hemodynamic stability [10], analgesic efficacy [11, 12], and decreased opioid-associated adverse effects (especially on postoperative nausea and vomiting) [13–15]. The application of OFA protocols could also address other effects of opioid use, such as addiction, development of pain chronicity, or even immunomodulation that may negatively impact postoperative infection or cancer [16]. Combining all these benefits may improve the overall quality of postoperative recovery (QoR) [11]. In addition, no data are available on longer-term outcomes.

We aim to perform a study to determine the impact of an OFA protocol versus standard of care (i.e., opioid use during anesthesia) on the QoR after major surgeries.

## Methods

**Fig. 1:**
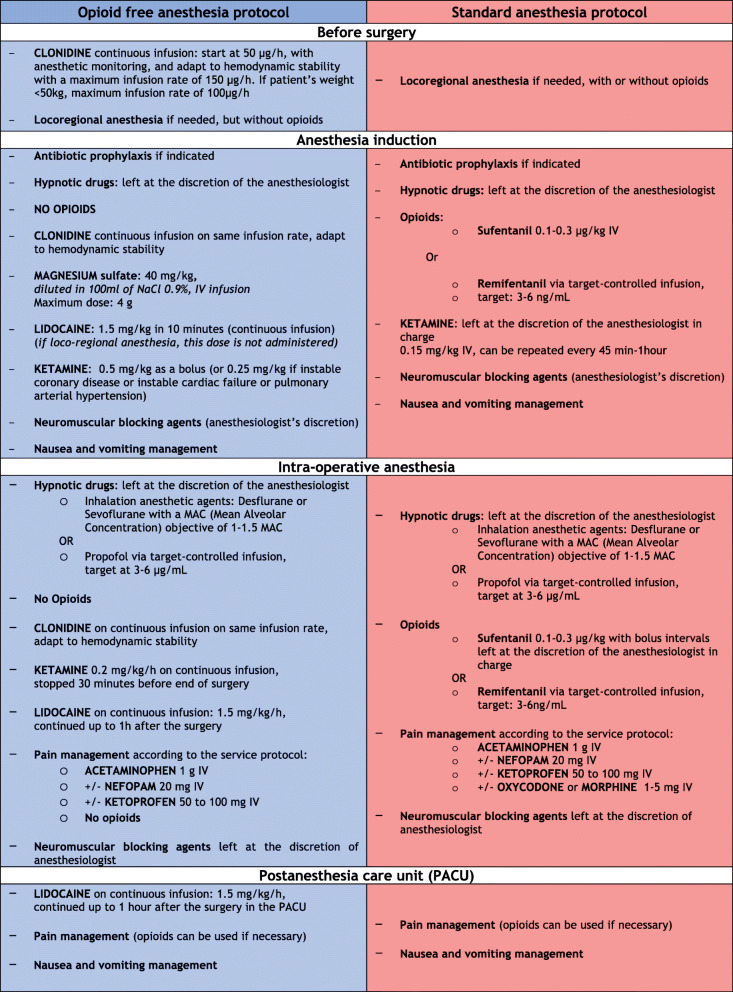
Detailed interventional protocols in the opioid-free anesthesia group (OFA group) and in the standard anesthesia group (English translation from our French procedures). IV, intravenous; PACU, postanesthesia care unit

**Fig. 2:**
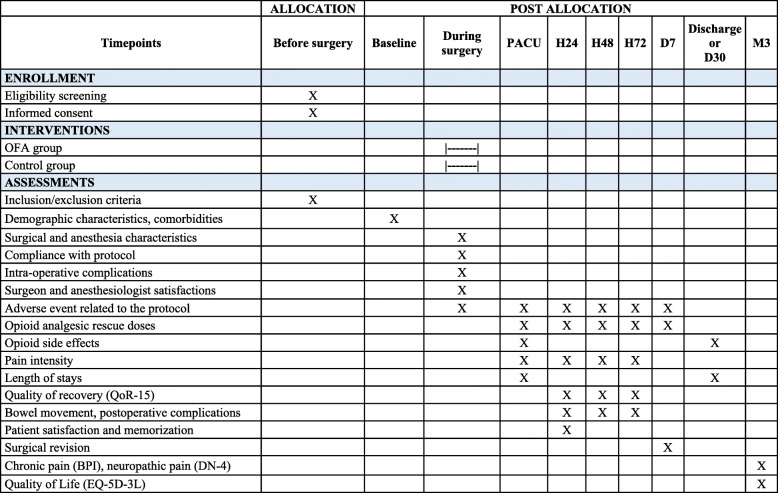
Standard Protocol Items: Recommendations for Interventional Trials (adapted from SPIRIT figure). BPI, Brief Pain Inventory; D7, 7 days after surgery; D30, 30 days after surgery; H24, 24 hours after surgery; H48, 48 hours after surgery; H72, 72 hours after surgery; M3, 3 months after surgery; OFA, opioid-free anesthesia; PACU, postanesthesia care unit

**Fig. 3:**
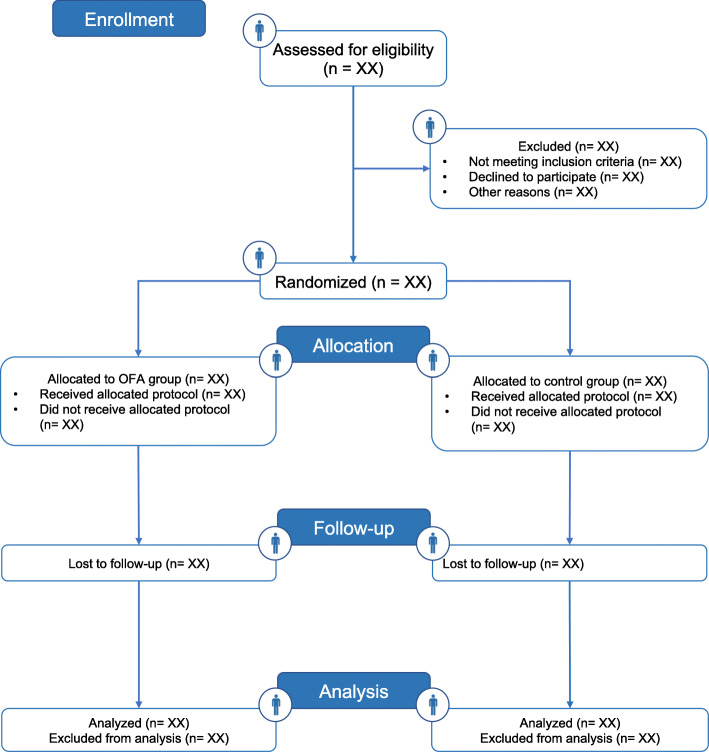
Flow chart

In this superiority, randomized, controlled, single-blinded, single-center trial, we plan to include patients scheduled to undergo elective major surgery (plastic, visceral, urologic, gynecologic, or ENT surgeries). The patients will be allocated to receive either an OFA protocol or a standard anesthesia protocol (Fig. 1). The study will be conducted at the Angers University Hospital, Angers, France. This trial is designed following the Standard Protocol Items (SPIRIT guidelines). Figures 2 and 3 provide an overview of the study plan.

The French Institutional Review Board “Eastern III” (Nancy, France, number: 2021-A00364-37) and the National Agency for Drug and Health Product Safety have approved the study protocol (2021-A00364-37) in its version 1. Patients will provide written consent for participation. The study will be conducted in accordance with the Declaration of Helsinki.

### Participants

Eligible patients are adults (age ≥ 18 years) undergoing a scheduled surgery with an estimated time of 90 min or more and supposed to be painful (with the usual need for postoperative intravenous (IV) morphine patient-controlled analgesia (PCA)). The surgical specialties concerned are ENT, plastic, digestive, urologic, and gynecologic surgery. Eligible patients must be French-speaking and must have sufficient cognitive ability to complete a questionnaire. The exclusion criteria are surgery with an orthopedic procedure (e.g., osteosynthesis), need for rapid sequence anesthetic induction, pregnancy or lactation, severe psychiatric or cognitive disorder interfering with questionnaire assessment, body mass index < 18 or > 39 kg/m^2^, any contraindication to study drugs, porphyria, uncontrolled epilepsy, unstable cardiac insufficiency, chronic renal or hepatic failure, preoperative bradycardia with an atrioventricular block (2nd or 3rd degree), chronic beta-blocker treatment, and ongoing treatment with a QT interval prolonging drug.

### Information of patients

During the anesthesia consultation, investigators will verify inclusion/exclusion criteria. The investigator will invite the patients to participate. Patients will receive complete information in faithful terms and understandable language concerning the objectives, the required follow-up, the risks, the safety measures, and the right to refuse to participate or stop the study at any time. The investigator obtained written informed consent signed by both the investigator and the patient.

### Randomization and blinding

The included patients will be randomized to one of the two groups (OFA or standard of care) using a web-based system (Ennov Clinical® software) by the investigator anesthesiologist in charge on the day or the day before the scheduled surgery.

Patients will be randomized in one of the two groups with a 1:1 ratio, using a minimization algorithm defined by the randomization methodologist (with a probabilistic component), based on two factors: the type of surgery (ENT, plastic, digestive, urologic and gynecologic surgery) and the surgery severity (assessed by the Surgical Outcome Risk Tool (SORT) score, which can be classified in minor, intermediate, major and major/complex surgery, and which is available online at www.sortsurgery.com). Investigators do not know this equation and will not be able to guess the next randomization arm.

The anesthesia team performing the general anesthesia will be unblinded on the allocation group. The patient will be kept blinded to the protocol allocation, as the nurses and the medical team involved in the postoperative care and outcomes evaluation. For outcomes requiring a phone call, evaluators will be blinded to the allocation group.

### Intervention

The study aims to compare an OFA protocol to an anesthesia protocol based on standard practices. In both arms, patients will undergo general anesthesia, combined or not with local or regional anesthesia. The two protocols are detailed in Fig. 1.

None of the patients will receive premedication. Anesthetic monitoring will be the same in both groups, including pulse oximetry, electrocardiography, non-invasive blood pressure and/or invasive monitoring when indicated, body temperature, muscle relaxation (train-of-four stimulation), and bispectral index.

The OFA protocol (OFA group) begins before surgery with a clonidine infusion (under cardiac monitoring) at an initial rate of 50 μg/h, and then adapted according to hemodynamic stability (to keep systolic blood pressure in a range of ± 20% its basal value) with a maximal rate of 150 μg/h (100 μg/h for patients with body weight < 50 kg). Anesthesia will be induced and maintained with hypnotic drugs and curare left at the anesthesiologist’s discretion. No opioids should be infused. The clonidine infusion should be continued at the same infusion rate. The OFA protocol includes a magnesium sulfate infusion (40 mg/kg), a lidocaine infusion (1.5 mg/kg bolus dose in 10 min followed by 1.5 mg/kg/h continuous infusion), and a ketamine infusion (0.5 mg/kg bolus dose followed by a 0.2 mg/kg/h continuous infusion). The ketamine infusion should be stopped 30 min before the end of the surgery, and the lidocaine infusion will be stopped after 1 h in the postanesthesia care unit (PACU).

The standard anesthesia protocol associates hypnotic drugs, curare, and opioids (sufentanil or remifentanil) left at the discretion of the anesthetist in charge. A low-dose ketamine bolus (0.15 mg/kg) is allowed at anesthesia induction, possibly followed by repeated boluses since it is commonly used in our standard practice.

In both arms, according to recent recommendations, anesthesiologists are prompted to infuse an antimicrobial prophylaxis [17], to apply a protective ventilation strategy (tidal volume between 6 and 8 ml/kg, respiratory rate for an end-tidal CO_2_ value at 35–40 mmHg, a positive end expiratory pressure) [18], and to treat perioperative hypotension and hypovolemia [19]. The depth of anesthesia is monitored by a bispectral index sensor (target 40–60). Postoperative pain, nausea, and vomiting are managed according to the unit protocols. The use of local or regional anesthesia is allowed in both groups (in the OFA group, the protocol stipulates not to use opioids as adjuvant). In the PACU, if the patient experiences acute pain (defined as a pain analog scale ≥ 4), morphine or oxycodone titration will be started (bolus of 1 to 2 mg, with a 5-min delay between two doses) until pain relief is achieved. The analgesic management will be continued in the ward using IV PCA and/or oral opioids. The doses of opioids (i.e., morphine and oxycodone) will be converted into morphine-equivalent doses according to the following rule: 1 mg oral morphine = 1/2 mg oral oxycodone = 1/3 mg IV morphine; 1 mg IV morphine = 1 mg IV oxycodone. Patients will be discharged to the ward when their modified Aldrete score is ≥ 12 [20].

### Outcomes

#### Primary outcome

The primary outcome is the early postoperative quality of recovery, assessed via the French version of the QoR-15 questionnaire score (FQoR-15) [21], and measured at 24 h after the surgery (postoperative day 1, POD 1). The QoR-15 score is currently the most reliable and reproducible tool to determine the quality of postoperative recovery [22–24]. Its use is recommended to assess the patient’s well-being, according to a recent international consensus [25] and has already been assessed by previous studies [26].

The FQoR-15 score is obtained via a self-administered questionnaire (which can also be performed through a phone interview) and consists in 15 items scored on an 11-point scale, with a total score (sum of each item) range from 0 to 150 (0 for very bad recovery and 150 for an excellent quality of recovery). The FQoR-15 questionnaire is given by the nurses in the ward on a paper sheet and is filled by the patient.

#### Secondary outcomes

In order to confirm the impact of the OFA protocol on postoperative recovery, we will also measure the QoR-15 score at 48 h and 72 h after the surgery in all patients. We will evaluate the pain during effort (mobilization, cough or physiotherapy sessions) and the number of postoperative complications according to the POMS (postoperative morbidity survey) classification [27], assessed up to 72 h after the surgery. The postoperative consumption of morphine-equivalent will also be compared between the two groups. The lengths of stay in PACU and in the hospital will be measured.

We will compare the patient tolerance to the allocated protocol with the assessment of the hemodynamic status (intra-operative use of vasopressor or cessation of one of the drugs for hemodynamic purposes), heart-rhythm (< 35 or > 140 beats per minute for more than 30 s), and anaphylactic reactions. Another secondary outcome is the respect of the allocated protocol by the anesthesia team: OFA protocol will be considered complete if at least two of the following drugs are used between ketamine, lidocaine, clonidine, and magnesium sulfate, and if no intraoperative opioids are used; the standard group will be considered complete if lidocaine, clonidine, or magnesium sulfate are not used intra-operatively. The surgeon’s and anesthesiologist’s satisfactions concerning the anesthesia will be assessed after the surgery according to a numerical scale (rated from 0 to 10). We will also compare the patient satisfaction of anesthesia at 24 h via a numerated scale from 0 to 10 (0 = not satisfied and 10 = totally satisfied) and the presence or absence of intra-operative memorization.

Three months after surgery, a phone interview will be conducted to evaluate the chronic pain using the Brief Pain Inventory (BPI) [28] in its French short version, the quality of life using the French EQ-5D-3 L questionnaire (available on request on https://euroqol.org/), and the rate of neuropathic pain diagnosed using the DN-4 questionnaire [29].

### Sample size

According to the database used to assess the French version of the QoR-15 that included 72 patients undergoing gynecologic, digestive, or urologic major surgery, the mean QoR-15 score was 97 at 24 h after the surgery, with a standard deviation of 16. A difference of at least 8.0 points in the score is considered clinically significant [30]. The OFA will thus be superior to the standard of care if the mean QoR-15 is at least 105. Considering a type I error risk of 0.05 in two-sided test and a power of 0.8, the estimated number of patients needed is 126 (63 patients in both groups). Considering that data will not be available for ten percent of the patients (e.g., surgery canceled, lost in follow-up, consent withdrawal) regarding the primary outcome, we plan to include 140 patients.

### Follow-up

Nine visits are scheduled for all patients enrolled in the study. The plan summarizing the follow-up visits is presented in Fig. 2. The time T0 (hour 0/day 0) is the time of the surgical incision. The following data will be recorded at inclusion: demographic data, weight and height, ASA physical status classification, surgery type, and comorbidities. The SORT classification will also be recorded.

Perioperative surgical and anesthetic data will be recorded, including total dose of each medication. The anesthesiologist and the surgeon will also grade their satisfaction concerning the anesthesia.

In PACU, the total dose of pain medications, the occurrence of any complication, including postoperative nausea and vomiting (PONV), numerical pain rating scale, and PACU length of stay (in hours) will be recorded. In the ward visits (at 24, 48, and 72 h after the surgery), we will record the following data: QoR-15 questionnaire, maximal numerical effort pain scale, the occurrence of complications (using the POMS classification), morphine consumption, bowel movement, adverse event, and patient satisfaction (only at 24 h after the surgery). The QoR-15 questionnaires will be completed by the patient himself whenever possible, who could be helped by a nurse blinded to the assignment group, or by phone if the patient has returned home.

At 7 days, we will collect the morphine or equivalent daily consumption and the occurrence of surgical revision. The following data will be recorded at the hospital discharged visit (or 30 days after surgery): total length of stay and occurrence of in-hospital opioid side effects. A phone call to the patient will be made at 3-month to record the BPI, the DN-4, and the EQ-5D scores.

### Safety

Safety items will be collected during follow-up by the anesthesia team (during surgery) and participating investigators during other visits. Various events will be collected during the anesthesia and surgery (e.g., episodes of arterial hypertension or hypotension, episodes of bradycardia, a suspected hypersensibility reaction, and surgical complications). The occurrence of other adverse events that may be attributable to the protocol will be collected during the other follow-up visits (at 24 h, 48 h, 72 h, 7 days, and until discharge (censored at 30 days)). The occurrence of a serious adverse event will be collected at any time during the patient's follow-up (up to 3 months), whose imputability with one of the allocation groups will be discussed. Details of safety monitoring are available in the supplementary material.

### Data collection and study monitoring

Data will be entered into the electronic web-based case report form (eCRF on Ennov Clinical®). We will establish the database from the eCRFs.

A clinical research associate (CRA) mandated by the study sponsor will ensure the successful completion of the study, the collection of data, documentation, recording, and report, in accordance with the Standard Operating Procedures implemented in the Angers Hospital and in accordance with Good Clinical Practice, laws, and regulations.

The following items will be reviewed for every fifteen included patients:

- Signed informed consent,
- Compliance with the study protocol and procedures that are defined,
- Quality of data collected in the case report form: accuracy, missing data, data consistency with the documents “source” (medical records, appointment books, original lab results, etc.).

After monitoring data on site, an automated check of the data entered will be made by the data management team based on the data validation plan signed by the coordinating investigator. Detected errors will lead to the issuance of requests for information and electronic correction.

### Statistical analysis

All analyses will be performed with R software (version 3.6.3). The main analysis will be in intention-to-treat and will include all randomized patients. Patients will be analyzed according to their randomization group. A per-protocol analysis will be performed as a sensitivity analysis to assess the main analysis robustness. A flow chart of all patients (Fig. 3) and descriptive statistics will be used to describe baseline characteristics. Data will be presented by their mean with standard deviation, median with interquartile range, number with the percentage of sample (%), or 95% confidence interval, according to their normal or non-normal distribution. We will report both absolute and relative measures for the comparisons between endpoints.

For the primary endpoint, we will use multivariate imputation for chained equations (with five imputations) via the mice package in R and perform a pooled-linear regression model, allowing us to perform an intention-to-treat analysis even if missing data. The covariates included in this analysis will be the allocation group, the type, and the surgery severity. This statistical method will also be used to analyze the QoR-15 at 48 and 72 h as secondary endpoints. To eliminate any influence of the phone evaluation, we will perform a sensitivity analysis just on the QoR-15 surveys collected in the ward.

We will also impute data for the analyses of other secondary endpoints: the rate of chronic pain patients at three months identified by the BPI via a logistic regression (same adjustment covariates) as well as for EQ-5D visual analog score via a linear regression (same adjustment covariates). The other endpoints will not be analyzed with the multiple imputation method. We reserve imputation for the endpoints included in the hierarchical scheme of the type I error management.

For each of the analyses involving the multiple imputation method, sensitivity analyses will be performed if the missing data rate for the endpoint of interest is greater than 10%. We will conduct an analysis only on the complete cases, as well as best-worst and worst-best case sensitivity analyses. For the best worst-case scenario, it is assumed that all participants lost to follow-up will have a beneficial outcome; and all those with missing outcomes in the other scenario will have a harmful outcome. For continuous outcomes, the beneficial outcome will be the group mean plus one standard deviation of the group mean, and the harmful outcome will be the group mean minus one standard deviation of the group mean [31]. To eliminate the potential risk of a violation of the normality, we will confirm the results for continuous variable implicating in the hierarchical scheme by sensitivity analysis using a semi-parametric approach with an ordinal model [32].

Concerning the pain evolution and morphine consumption variation, we will analyze those outcomes using a mixed effects linear regression model (fixed effect for the treatment arm, a fixed effect for the time frame, a fixed effect for each minimization factor, and a random effect for the patients). Hospital and PACU length of stays, as well as satisfactions (those of the surgeon, those of the anesthesiologist, those of the patient), will be compared using a linear regression model (allocation group and minimization factors as covariates). The proportion of complications of the POMS will be defined as a categorical variable (four levels: no complication, one complication, two complications, and more than 3 complications) and will be analyzed using a logistic regression model (allocation group and the minimization factors as covariates). The rate of patients presenting neuropathic pain, as well as the proportion of patients experiencing memorizations, will be compared by logistical regression models (allocation group and the minimization factors as covariates). Pain characteristics described by BPI and the proportion of patients in each of the five scales of EQ-5D will not be compared but only described.

All statistical analyses will be performed by two-sided tests. The signification cutoff will be a *p* value < 0,05 with 95% confidence interval.

### Type I error management

**Fig. 4:**
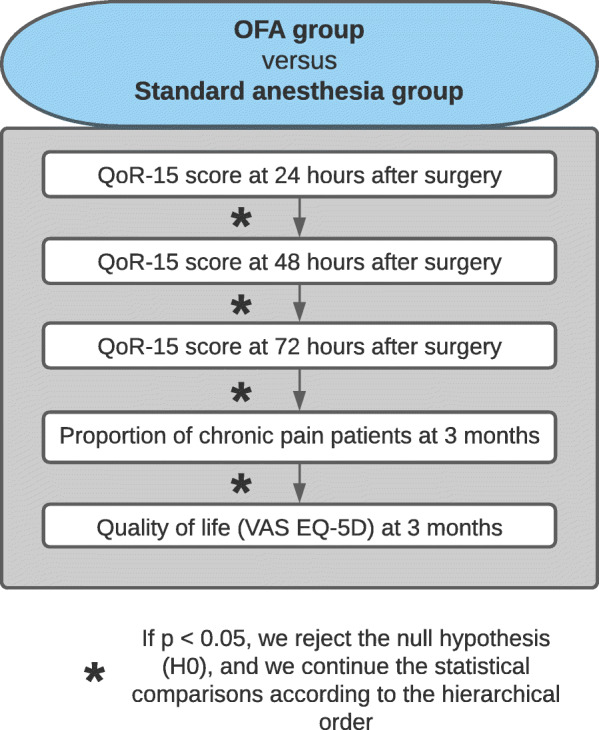
Diagram of the hierarchical classification of the statistical tests (type I error management). OFA, opioid-free anesthesia; VAS, visual analog scale

We will manage the type I error in a sequential hierarchical manner (Fig. 4). We will test the first endpoint (i.e., QoR-15 at H24), and if the *p* value is less than 0.05, we will conclude that there is evidence of an effect. Then, we will test the following endpoint (i.e., QoR-15 at H48) using a significance level of 0.05. We will continue to test each endpoint of the hierarchical classification either up to the last endpoint (i.e., the quality of life by the EQ-5D visual analog scale at 3 months) or if we observe a non-significant result. Suppose a non-significant result (*p* value > 0.05) for one of the endpoints is observed. In that case, we will perform the following statistical comparisons in an exploratory manner without drawing any clinical conclusion.

No interim analysis is planned during the inclusion period.

## Discussion

Although OFA seems promising in the context of the opioid crisis, data are needed to objective its interest; our study will be powered to evaluate the impact of OFA on early QoR and give useful information on long-term benefit of reducing perioperative opioid use. In addition, we choose to use a combination of lidocaine, magnesium sulfate, ketamine, and clonidine to perform OFA. Other studies are underway which attempt to evaluate the quality of postoperative recovery using OFA, but either do not fully assess this “full” combination of drugs [33], or do not propose a continuous infusion throughout surgery [34]. In addition, we plan to evaluate OFA in a wide range of surgeries, while other studies focus on one type of surgical procedure only, in order to provide more generalizable results.

We choose to combine these different pharmacological classes during surgery for their different effects: analgesia, anti-hyperalgesia, anti-inflammatory effect, anti-nausea and anti-vomiting, and reduction of the neurohumoral stress response. In the anesthesia literature, different OFA protocols were described [35]. While OFA using dexmedetomidine has been reported to be effective during several types of surgery [13, 36], it may promote side effects, including increased risk of bradycardia and hypoxemia [9]. We thus prefer to incorporate clonidine as an alpha-2 agonist agent in our OFA protocol, because this molecule seems to be less responsible for severe bradycardia episodes [37] and seems to have longer positive effects [38].

To evaluate OFA, we choose primary criteria focused on patients’ overall recovery. Indeed, in a recent international consensus, the SteP–COMPAC group has highlighted the value of postoperative recovery scales for standardizing outcomes in perioperative medicine [25]. The QoR-15 questionnaire appears reliable, sensitive, and easily achievable in clinical practice (2 to 3 min for the questionnaire to be fully completed). It gives information on postoperative recovery in a unidimensional approach, and the patient can complete the questionnaire alone or with the help of an assessor. Therefore, different studies have used this questionnaire to evaluate the impact of an intervention on patients’ perception of postoperative recovery [26]. Beyond assessing the quality of early recovery, we will also study the longer-term status (i.e., at 3 months). The study aims to investigate the pain profile of the patients, detect the presence of chronic pain, neuropathic pain, and estimate their quality of life. To our knowledge, our study would be the first to evaluate OFA on these long-term criteria.

In a nutshell, this study will compare an OFA protocol (based on a combination of four agents: clonidine, ketamine, magnesium sulfate, and lidocaine) versus a standard anesthesia protocol in patients scheduled for major non-orthopedic surgery, on their early QoR but also on their pain status and their quality of life at 3 months. The findings will also provide evidence of potential adverse events associated with this OFA protocol, helping to assess the benefit/risk ratio of the use of such a pharmacological combination.

## Trial status

This study was approved by the French Institutional Review Board “Eastern III” (Nancy, France, number: 2021-A00364-37) and the National Agency for Drug and Health Product Safety, and registered at ClinicalTrials.gov (NCT04797312) on 15 March 2021. The recruitment of participants started in July 2021. The anticipated period is 18 months.

## Data Availability

The data generated could be shared after a request to the corresponding author. Three years after the end of follow-up of the last included patient, we will deliver a completely deidentified dataset to an appropriate data archive for sharing purposes.

## Abbreviations

BPI: Brief Pain Inventory
ENT: Ear, nose, and throat
EQ-5D: Euroqol-5D score
IV: Intravenous
OFA: Opioid-free anesthesia
PACU: Post anesthesia care unit
PCA: Patient-controlled analgesia
POMS: Postoperative morbidity survey
PONV: Postoperative nausea and vomiting
QoR: Quality of recovery
SORT: Surgical Outcome Risk Tool
